# Continuous Elevation of PTH Increases the Number of Osteoblasts via Both Osteoclast-Dependent and -Independent Mechanisms

**DOI:** 10.1002/jbmr.145

**Published:** 2010-06-07

**Authors:** Robert L Jilka, Charles A O'Brien, Shoshana M Bartell, Robert S Weinstein, Stavros C Manolagas

**Affiliations:** Division of Endocrinology & Metabolism, Center for Osteoporosis and Metabolic Bone Diseases, Central Arkansas Veterans Healthcare System, University of Arkansas for Medical Sciences Little Rock, AR, USA

**Keywords:** parathyroid hormone, sRANKL, osteoblasts, osteoclasts, bone remodeling, hyperparathyroidism

## Abstract

Sustained parathyroid hormone (PTH) elevation stimulates bone remodeling (ie, both resorption and formation). The former results from increased RANKL synthesis, but the cause of the latter has not been established. Current hypotheses include release of osteoblastogenic factors from osteoclasts or from the bone matrix during resorption, modulation of the production and activity of osteoblastogenic factors from cells of the osteoblast lineage, and increased angiogenesis. To dissect the contribution of these mechanisms, 6-month-old Swiss-Webster mice were infused for 5 days with 470 ng/h PTH(1-84) or 525 ng/h soluble RANKL (sRANKL). Both agents increased osteoclasts and osteoblasts in vertebral cancellous bone, but the ratio of osteoblasts to osteoclasts and the increase in bone formation was greater in PTH-treated mice. Cancellous bone mass was maintained in mice receiving PTH but lost in mice receiving sRANKL, indicating that maintenance of balanced remodeling requires osteoblastogenic effects beyond those mediated by osteoclasts. Consistent with this contention, PTH, but not sRANKL, decreased the level of the Wnt antagonist sclerostin and increased the expression of the Wnt target genes *Nkd2, Wisp1*, and *Twist1*. Furthermore, PTH, but not sRANKL, increased the number of blood vessels in the bone marrow. Weekly injections of the RANKL antagonist osteoprotegerin at 10 µg/g for 2 weeks prior to PTH infusion eliminated osteoclasts and osteoblasts and prevented the PTH-induced increase in osteoclasts, osteoblasts, and blood vessels. These results indicate that PTH stimulates osteoclast-dependent as well as osteoclast-independent (Wnt signaling) pro-osteoblastogenic pathways, both of which are required for balanced focal bone remodeling in cancellous bone. © 2010 American Society for Bone and Mineral Research.

## Introduction

During bone remodeling, old bone is removed and replaced with new by teams of osteoclasts and osteoblasts, collectively called the *basic multicellular unit* (BMU). In cancellous bone, the BMU is separated from the marrow by a canopy of flat cells in close association with blood vessels lying just outside the canopy.(1,2) Hypoparathroidism and hyperparathyroidism lead to a decrease or increase in the number of BMUs, respectively, consistent with a major role of parathyroid hormone (PTH) in the rate of bone remodeling.(3,4) A remarkable feature of PTH-regulated remodeling in cancellous bone is that the number of osteoblasts recruited to the BMU is sufficient to completely refill the resorption cavity. Indeed, cancellous bone is maintained or increases in hyperparathyroidism,(5) while cortical bone is lost in this condition.

PTH initiates new BMUs by promoting the differentiation of osteoclasts from circulating hematopoietic progenitors. This effect is mediated by a stimulatory effect of the hormone on the synthesis of receptor activator of NF-κB ligand (RANKL)(6,7)—a membrane-bound cytokine that binds to RANK on osteoclast progenitors and stimulates their differentiation. The pro-osteoclastogenic effect of PTH is intensified by its ability to suppress the production of osteoprotegerin (OPG), a soluble decoy receptor for RANKL. The importance of PTH-stimulated RANKL synthesis for the control of the rate of bone remodeling is highlighted by evidence that deletion of a PTH-responsive enhancer of RANKL gene transcription attenuates expression of this cytokine and reduces the rate of bone remodeling in adult mice.(8)

Several mechanisms have been proposed to explain the coupling of bone formation to bone resorption within the BMU. One of the mechanisms is the release of factors from the calcified bone matrix during bone resorption, such as insulin-like growth factors (IGFs), bone morphogenetic proteins (BMPs), and transforming growth factor β (TGF-β),(9–11) that stimulate osteoblast differentiation. Osteoclasts themselves also produce several pro-osteogenic agents, including cardiotropin 1,(12) sphingosine-1-phosphate, and BMP-6.(13) The existence of an osteoclast-mediated pro-osteoblastogenic mechanism is supported by evidence that administration of soluble RANKL (sRANKL) to rats or mice increases serum markers of both bone formation and bone resorption.(14,15) PTH also acts on cells of the osteoblast lineage, including osteocytes, to affect the expression and activity of a variety of pro-osteoblastogenic growth factors.(16–18) PTH stimulation of canonical Wnt signaling, which is essential for osteoblast differentiation,(19) may play a particularly prominent role. Activation of the PTH receptor in osteoblastic cells directly induces canonical Wnt signaling,(20) and suppression of the synthesis of the Wnt antagonist sclerostin by osteocytes may intensify this pro-osteoblastogenic signal further.(21,22) The powerful role of sclerostin-mediated inhibition of bone formation is highlighted by the dramatic increase in bone formation in ovariectomized rats following administration of an antisclerostin antibody.(23,24)

PTH also may regulate osteoblast differentiation via effects on angiogenesis because bone formation during development, fracture healing,(25) and remodeling(1,2) almost always takes place in close proximity to capillaries. The linkage of angiogenesis and osteoblastogenesis may be due in part to the fact that mesenchymal stem cell progenitors of osteoblasts are located on the outside surfaces of blood vessels.(26) Furthermore, PTH stimulates the production of angiopoetin 1 by osteoblastic cells(27) and of vascular endothelial growth factor (VEGF) by endothelial cells;(28) and vascular endothelial cells produce a variety of pro-osteoblastogenic factors.(25)

Here we examined the potential contribution of these three pathways to the increase in osteoblasts caused by continuous elevation of PTH. We show in mice that infusion of PTH stimulates balanced focal remodeling of cancellous bone, whereas infusion of sRANKL causes unbalanced remodeling and bone loss. Hence the osteoclast-dependent pro-osteoblastogenic mechanism, in and of itself, does not generate a sufficient number and quality of osteoblasts to refill the resorption cavity. We also show that PTH, but not sRANKL, stimulates Wnt signaling as well as angiogenesis. Interestingly, pretreatment of mice with OPG-Fc blocks both the pro-osteoblastogenic and the proangiogenic effects of the hormone. We conclude that PTH stimulates osteoclast-dependent and -independent (Wnt signaling) pro-osteoblastogenic pathways, both of which are required for balanced focal bone remodeling in cancellous bone.

## Materials and Methods

### Animals

Female Swiss-Webster mice (6 months old, 29 to 31 g) were obtained from Harlan (Indianapolis, IN, USA) and were maintained and used in accordance with NIH guidelines. Animal use protocols were approved by the Institutional Animal Care and Use Committees of the University of Arkansas for Medical Sciences and the Central Arkansas Veterans Healthcare System. Mice were fed a standard rodent diet (Teklad 22/5, Harlan) containing 22% protein, 1.13% calcium, and 0.94% phosphorus. Mice were injected every 7 days with PBS (vehicle) or 10 µg/g of human OPG-Fc (provided by Paul Kostenuik, Amgen, Seattle, WA, USA). After 14 days of this pretreatment, mice were infused for 5 days with 0.001% β-mercaptoethanol, 0.06% acetic acid (PTH vehicle), 470 ng/h PTH(1-84) (Bachem, Torrance, CA, USA), or 525 ng/h recombinant human sRANKL (provided by Paul Kostenuik, Amgen) dissolved in PBS using microosmotic pumps delivering fluid at 1 µL/h (Durect Corp., Cupertino, CA, USA). Pumps were implanted into an interscapular subcutaneous pocket under anesthesia, as described previously.(29) Tetracycline (30 mg/kg, i.p.) was given 2 days prior to beginning the infusion, and alizarin red (30 mg/kg, i.p.) was given 2 days before the end of the experiment to permit determination of the bone-formation rate. Serum was obtained by retroorbital bleeding for measurement of osteocalcin (Biomedical Technologies, Soughton, MA) at the end of the experiment.

### Micro–computed tomography (µCT)

µCT analysis of femurs and lumbar vertebrae (L_4_) was done after the bones were dissected, cleaned, fixed in 10% Millonig's formalin, and transferred to ethanol. Bones were imaged by µCT (µCT40, Scanco Medical, Basserdorf, Switzerland). Scans were integrated into 3D voxel images (1024 × 1024 pixel matrices for each individual planar stack). A Gaussian filter (σ = 0.8, support = 1) was used to reduce signal noise, and a threshold of 200 was applied to all analyzed scans. Scans were done at 12-µm resolution (*E* = 55 kVp, *I* = 145 µA, integration time = 200 ms). All trabecular measurements were made by manually drawing contours every 10 to 20 slices and using voxel counting for determination of bone volume per tissue volume. Calculation of trabecular microarchitecture was done using sphere-filling distance-transformation indices without assumptions about the bone shape as a rod or plate.

### Histomorphometry

After fixation in Millonig's formalin, lumbar vertebrae (L_1_–L_3_) were embedded nondecalcified in methyl methacrylate by an automated procedure using a Tissue-Tek-VIP machine (Sakura Finetek, Torrance, CA, USA). Histomorphometric analysis of bone sections and detection of apoptotic osteoblasts by in situ nick-end labeling (ISEL) were performed as described previously.(30) Measurements of cancellous bone were made in the entire secondary spongiosa of all three vertebrae. Osteoblasts were recognized by their juxtaposition to osteoid and counted only if seen in groups of two or more. Osteoclasts were identified by TRACPase staining. The bone-formation rate was calculated by multiplying the mineral apposition rate by the linear extent of the double-labeled perimeter. Because the tetracycline was given prior to initiating the infusion protocol, changes in double-labeled surfaces reflect actions of PTH and sRANKL on existing BMUs. The alizarin red–labeled perimeter was used as an index of osteoblast activity during the infusion period. Variables were measured and reported as described previously(30) using terminology recommended by the Histomorphometry Nomenclature Committee of the ASBMR.(31)

Blood vessels in the marrow space adjacent to the secondary spongiosa were recognized by their content of red blood cells and presence of CD34^+^ endothelial cells. For the latter, nondecalcified bone sections were deplasticized with 2-methoxyethyl acetate and then sequentially rehydrated in 95%, 80%, and 70% ethanol and then water. After rinsing in PBS, antigen retrieval was performed in a programmable histology microwave oven heated to 48°C for 1 minute in 0.01 M Na-citrate, pH 6, followed by incubation in 0.05% pepsin in 0.1 N HCl for 30 minutes at 37°C. After rinsing, sections were quenched with 3% H_2_O_2_ in methanol and blocked with diluted normal mouse serum. Sections were incubated overnight at 4°C with a 1:50 dilution of mouse monoclonal anti-CD34 antibody (Vector Laboratories, Inc., Burlingame, CA, USA). After rinsing, bound antibody was visualized with a peroxidase-labeled secondary antibody (Vector Laboratories, Inc.) according to the manufacturer's instructions.

### Western blotting

Sclerostin in lysates prepared from tibias was quantified by Western blotting as described previously,(21) except that 20 µg of protein per lane was applied. Antisclerostin was from R&D Systems (Minneapolis, MN, USA), anti–mouse β-actin was from Sigma (St. Louis, MO, USA). Goat anti–mouse horseradish peroxidase was from Santa Cruz Biotechnology (Santa Cruz, CA, USA). Peroxidase labeling was detected using SuperSignal West Pico Chemiluminescent Substrate (Pierce, Rockford, IL, USA). Chemiluminescence was quantified with a VersaDoc Image System (Model 3000) and QuantityOne software (Biorad, Hercules, CA, USA).

### Transient transfection and luciferase assay

pcDNA was purchased from Invitrogen (Carlsbad, CA, USA). A reporter plasmid carrying three Tcf binding sites upstream of a minimal c-fos promoter driving the firefly *Luciferase* gene (TOPFLASH)(32) was provided by B Vogelstein (Johns Hopkins University Medical Institutions, Baltimore, MD, USA). Osteoblastic UAMS-32P cells(33) or UMR 106 cells were transfected with the reporter plasmid using Lipofectamine Plus (Invitrogen). *Luciferase* activity was determined 24 hours later using the Dual-Luciferase Reporter assay system (Promega, Madison, WI, USA). Light intensity was measured with a luminometer, and the *Luciferase* activity was divided by the *Renilla* activity (control reporter) to normalize for transfection efficiency.

### Quantitative PCR

RNA was extracted from lumbar vertebrae (L_5_) as described previously.(21) Transcripts were quantified by TaqMan PCR using Assay-on-Demand primer and probe sets from Applied Biosystems (Foster City, CA, USA).

### Statistics

Data were analyzed by Student's two-tailed *t* test or by ANOVA using SigmaPlot and SigmaStat (SPSS Science, Chicago, IL, USA). Values are reported as the mean ± SD. If necessary, data were log-transformed to achieve a normal distribution. For ANOVA, *p* values were Bonferroni-adjusted for the relevant number of post hoc comparisons to control the overall error rate.

## Results

Bone remodeling in female Swiss-Webster mice (6 months old) was stimulated by infusing PTH(1-84) at 470 ng/h for 5 days using an osmotic pump. We have shown previously that this dose of PTH and duration of infusion increase osteoclast and osteoblast number,(30) but peritrabecular fibroblasts, which are thought to be arrested preosteoblasts,(34) have not yet appeared. The magnitude of the increase in osteoblast number thus reflects PTH-regulated events that govern recruitment of osteoblasts to the BMU in the absence of confounding effects of chronic hyperparathyroidism. To determine the specific contribution of osteoclast-dependent mechanisms of PTH-stimulated bone formation, mice were infused for 5 days with recombinant human sRANKL at 525 ng/h. This dose was shown previously to increase serum levels of TRACPase and osteocalcin after 3 or 7 days of administration in rats.(14)

**Fig. 1:**
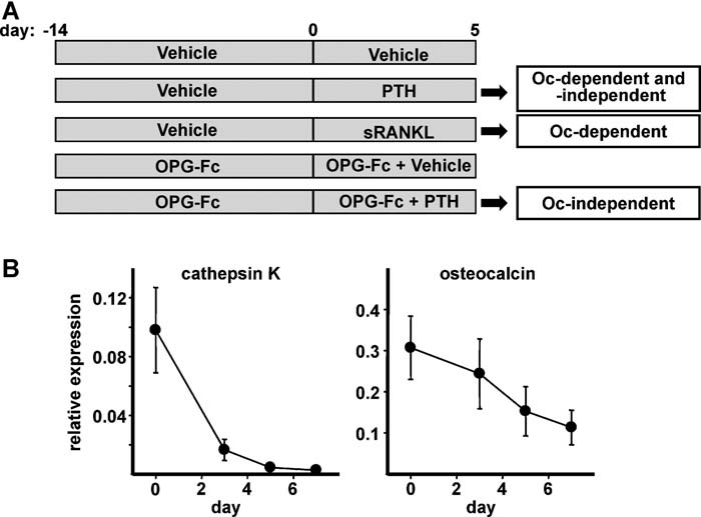
Experimental design. (*A*) Female Swiss Webster mice were treated as indicated to distinguish osteoclast (Oc)–dependent and –independent mechanisms for increasing osteoblast number during PTH-stimulated bone remodeling. (*B*) The decline in osteoclasts and osteoblasts in vertebral bone (L_5_) following a single injection of 10 µg/g of OPG-Fc on day 0 was monitored using quantitative PCR (qPCR) at the indicated times to determine expression of the osteoclast-specific gene *cathepsin K* and the osteoblast-specific gene *osteocalcin*, relative to GAPDH (*n* = 6 to 8/group).

The experimental design is schematically depicted in Fig. 1*A*. To investigate whether PTH can stimulate osteoblast differentiation solely via osteoclast-independent mechanisms, bone was depleted of osteoclasts by injecting mice with 10 µg/g of OPG-Fc, a long-lasting form of OPG,(14,15) at 14 and 7 days prior to infusion of PTH. The OPG-Fc pretreatment protocol is based on results from a preliminary experiment showing that a single injection of OPG-Fc caused a rapid decline in osteoclasts followed by a slower decline in osteoblasts, as reflected by expression of *cathepsin K* and *osteocalcin* mRNA, respectively (Fig. 1*B*). The slower decline in osteoblasts reflects their 10- to 14-day lifespan,(35) during which time they elaborate a collagenous matrix and then die by apoptosis or become osteocytes or lining cells. Two weeks of OPG-Fc administration achieved continued suppression of osteoclastogenesis and complete depletion of osteoblasts prior to infusion of PTH (data not shown).

Body weight was not affected by any of the treatments (Table 1). PTH, but not sRANKL, increased total serum Ca, consistent with a previous report.(14) The PTH-stimulated increase in serum calcium was abrogated by pretreatment with OPG-Fc, reflecting the blockade of RANKL and thereby the increased bone resorption caused by PTH (Table 1).

**Table 1:** Changes in Body Weight and Total Serum Calcium

Treatment	Body weight, % change	Ca, mg/dL
Vehicle	0.8 ± 6.8	9.8 ± 1.4
PTH(1–84)	2.8 ± 11.0	13.0 ± 1.3*
sRANKL	3.1 ± 6.6	9.6 ± 2.2
OPG-Fc	3.8 ± 3.6	9.9 ± 2.0
OPG-Fc + PTH(1–84)	3.2 ± 3.8	10.6 ± 2.9

*Note: n* = 9 to 12/group.

* *p* < .05 versus vehicle.

### sRANKL, but not PTH, causes loss of vertebral cancellous bone

**Fig. 2:**
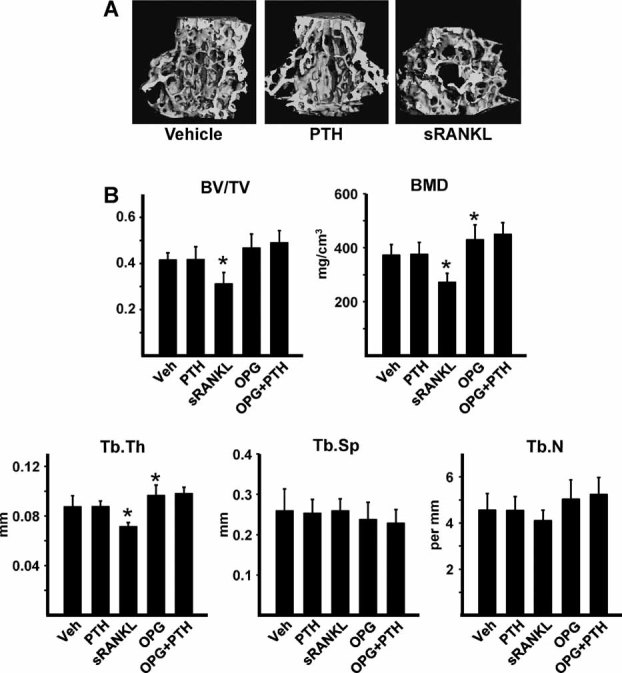
sRANKL, but not PTH, decreases vertebral cancellous bone mass. Vertebral bone (L_4_) was analyzed by µCT. (*A*) Representative µCT images. (*B*) Measurements of cancellous bone volume per tissue volume (BV/TV), BMD, trabecular thickness (Tb.Th), trabecular separation (Tb.Sp), and trabecular number (Tb.N). *n* = 8 to 10/group. *p < .05 versus vehicle (Veh) control.

**Fig. 3:**
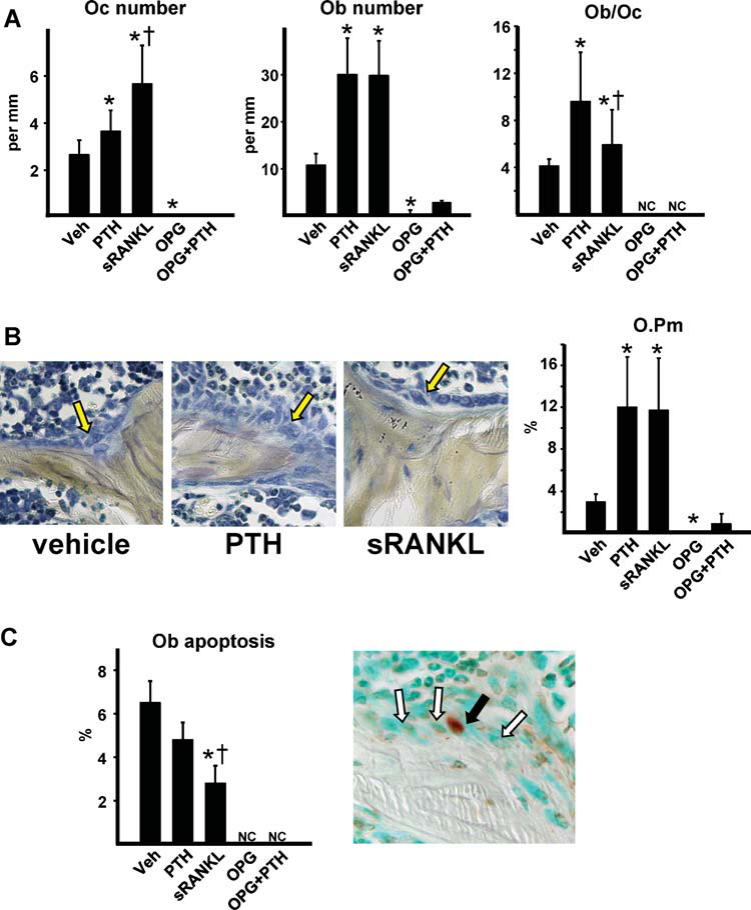
Reduced osteoblast number in response to sRANKL compared with PTH and blockade of PTH effects by OPG-Fc. (*A*) Histomorphometric determination of osteoclast (Oc) and osteoblast (Ob) numbers was done in vertebral cancellous bone sections (L_1_–L_3_). Owing to the severe reduction in osteoblasts and osteoclasts in bone of mice pretreated with OPG, the ratio of Ob to Oc was not calculated (*NC*) in these specimens. (*B*) Photomicrographs of osteoblasts (*arrows*) in animals receiving vehicle, PTH, or sRANKL. Nondecalcified sections, original magnification ×400. Osteoid perimeter (O.Pm) was determined as in panel *A*. (*C*) Prevalence of osteoblast apoptosis as determined by ISEL. Osteoblast apoptosis was not calculated (*NC*) in mice pretreated with OPG owing to the very low number of osteoblasts available for inspection. The photomicrograph shows an apoptotic osteoblast (*black arrow*) among a group of viable osteoblasts (*white arrows*) in a bone section from a sRANKL-treated mouse. *n* = 4 to 6/group. *p < .05 versus vehicle (Veh) control; ^†^*p* < .05 versus PTH.

µCT measurements indicated that PTH had no effect on the cancellous architecture of lumbar vertebrae (L_4_) (Fig. 2). In contrast, sRANKL caused a 25% reduction in cancellous bone volume and in bone mineral density (BMD). The decreased bone volume was due to reduced trabecular thickness, consistent with unbalanced focal remodeling. Trabecular separation and trabecular number were unaffected by sRANKL. Thus osteoclastic perforation of individual trabecular elements did not occur. The reduced trabecular thickness was not associated with an increase in trabecular spacing. This is so because the spacing measurement employs skeletonized images of trabecular profiles; that is, the measurement is made from the middle of each trabecular element rather than the edge. OPG-Fc administration increased trabecular thickness and BMD, most likely owing to depletion of osteoclasts and the refilling of existing resorption cavities by osteoblasts during the 19 days of treatment with this agent (Fig. 3*A*).

Cortical thickness at the femoral diaphysis was unaffected by PTH, sRANKL, or OPG-Fc (not shown). A high coefficient of variation prevented detection of effects of the treatments on cancellous bone at the distal femur (not shown). Hence the remainder of the study was focused on vertebral cancellous bone.

### The bone remodeling stimulated by sRANKL is characterized by lower osteoblast number and activity compared with PTH

Histomorphometric measurements done on nondecalcified sections of vertebral cancellous bone showed that the increase in osteoclast number caused by sRANKL was approximately twofold greater than that caused by PTH (Fig. 3*A*). In contrast, bone from animals receiving sRANKL or PTH exhibited an equivalent increase in the number of osteoblasts. Importantly, therefore, the ratio of osteoblasts to osteoclasts was greater in PTH-treated mice than in sRANKL-treated mice (Fig. 3*A*). In other words, for a given increase in osteoclasts, the corresponding rise in osteoblast number was greater in mice infused with PTH than in mice given sRANKL.

In both PTH- and sRANKL-treated animals, osteoblasts exhibited typical plump cuboidal morphology adjacent to osteoid, similar to that seen in vehicle-treated animals (Fig. 3*B*). Accordingly, and consistent with the increase in osteoblast number, osteoid surface was increased by both PTH and sRANKL (Fig. 3*B*). Peritrabecular fibrosis was not observed in any of the bone specimens. As we reported previously,(30) the prevalence of apoptotic osteoblasts was not affected by infusion of PTH (Fig. 3*C*). Strikingly, however, sRANKL caused a reduction in the prevalence of apoptotic osteoblasts.

**Fig. 4:**
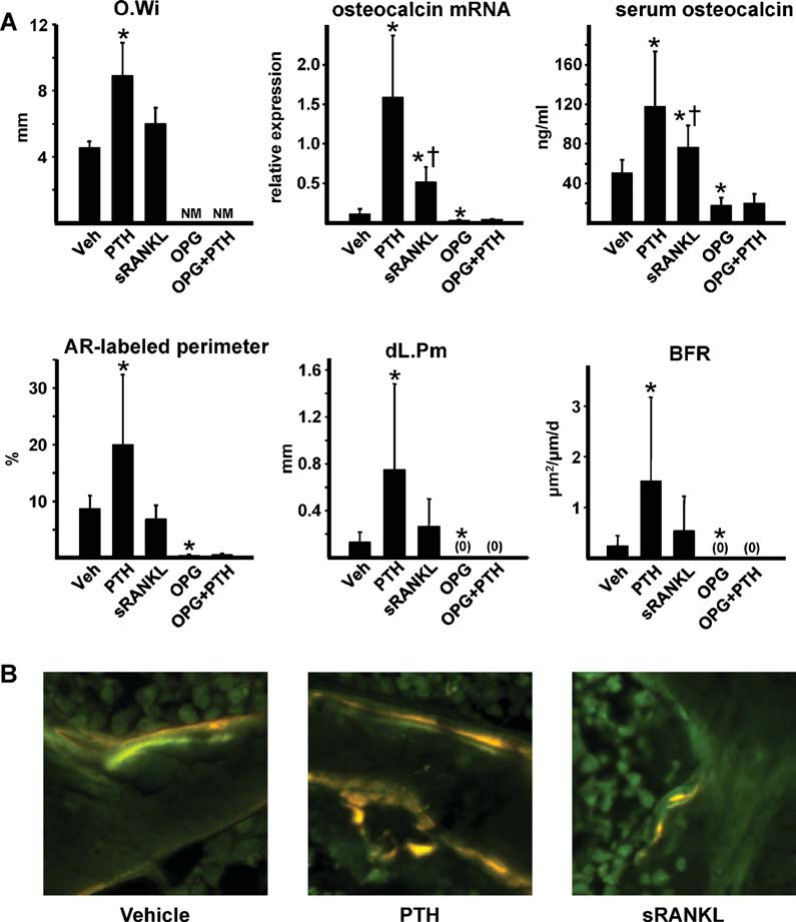
Reduced osteoblast activity in mice receiving sRANKL compared with PTH and blockade of PTH effects by OPG-Fc. (*A*) Histomorphometric determination of osteoid width (O.Wi), alizarin red (AR)–labeled perimeter, double-labeled surface (dL.Pm), and bone-formation rate (BFR) using the same sections as in Fig. 3. Osteocalcin transcripts were measured by qPCR of RNA preparations from vertebral bone (L_4_). Serum osteocalcin was measured by RIA. Osteoid width was not measured (*NM*) in mice pretreated with OPG-Fc. *n* = 4 to 6/group. *p < .05 versus vehicle (Veh) control; ^†^*p* < .05 versus PTH. (*B*) Double labeling with tetracycline and alizarin red in vertebral bone sections from mice infused with vehicle, PTH, or sRANKL. Original magnification ×400.

Functional analysis indicated that the osteoblasts that develop in response to sRANKL had lower bone-formation capacity than those that develop in response to PTH. Specifically, PTH increased osteoid width, but sRANKL did not (Fig. 4*A*). Despite the fact that PTH and sRANKL caused a similar increase in osteoblast number, the increase in osteocalcin transcripts in vertebral bone of sRANKL-treated mice was approximately one-third that seen in PTH-treated mice. Moreover, PTH caused a much greater increase in serum osteocalcin than sRANKL.

PTH increased bone formation, as indicated by alizarin red labeling (given on day 3 of the experiment), whereas sRANKL had no effect (Fig. 4*A*). Tetracycline was given 2 days prior to initiation of infusion to permit determination of the effects of PTH and sRANKL on bone formation within existing BMUs by measuring the double-labeled perimeter. Neither agent affected mineral apposition rate (not shown). However, PTH, but not sRANKL, increased the double-labeled perimeter and the bone-formation rate (Fig. 4*A, B*). The failure to observe an sRANKL-stimulated increase in bone-formation rate in the face of increased osteocalcin and osteoid perimeter in this study may be due to the fact that alizarin red labeling reflects osteoblast activity at 3 days after beginning infusion of PTH or sRANKL, whereas the other indices reflect osteoblast activity at the end of the experiment (ie, after 5 days of infusion). These findings indicate that bone remodeling stimulated by sRANKL is unbalanced in favor of bone resorption owing to inefficient generation of osteoblasts with enough functional capacity to completely refill the resorption cavity.

### OPG prevents the PTH-stimulated increase in osteoblasts

Vertebral bone sections from mice receiving OPG-Fc for 14 days prior to infusion of vehicle for 5 days exhibited few, if any, osteoclasts or osteoblasts (Fig. 3*A*). As expected, OPG administration prior to PTH infusion prevented the increase in osteoclast number. More important, PTH failed to significantly increase osteoblast number in mice pretreated with OPG-Fc, albeit a few osteoblasts overlying osteoid were noted in two of the six bone sections. The very low number of osteoblasts precluded accurate determination of the prevalence of apoptotic osteoblasts in mice receiving OPG-Fc.(36) Osteocalcin mRNA expression and circulating osteocalcin, as well as labeling with alizarin red, were sharply reduced in OPG-Fc-pretreated mice. Under these conditions, PTH was unable to increase these measures of osteoblast number and function (Fig. 4*A*). No double-labeled surface was detected in mice receiving OPG-Fc, without or with subsequent PTH infusion (Fig. 4*A*). Taken together, these findings demonstrate that the increase in osteoblasts caused by continuous elevation of PTH cannot occur in the absence of BMUs.

### PTH, but not sRANKL, attenuates sclerostin expression and activates Wnt signaling

**Fig. 5:**
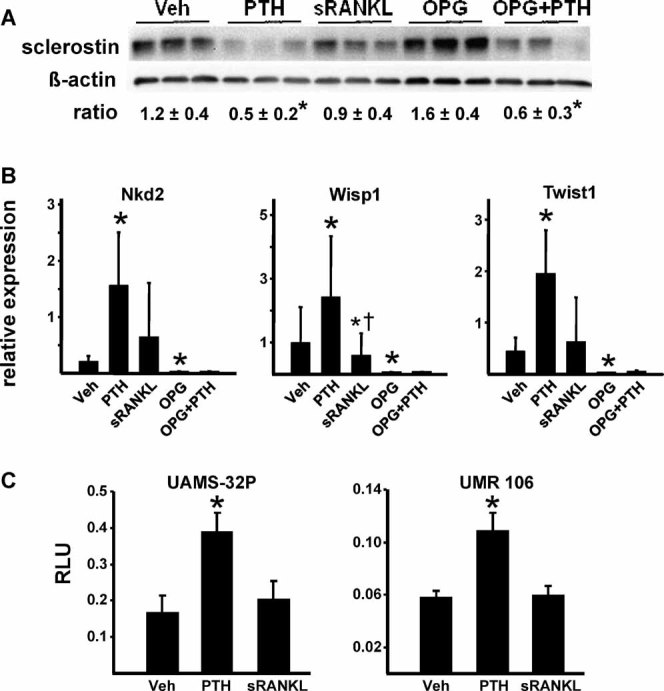
PTH, but not sRANKL, attenuates sclerostin expression and activates Wnt signaling. (*A*) Sclerostin was extracted from tibias and quantified by Western blotting. Each lane represents one animal. Blots of three of the six samples in each group are shown. *p < .05 versus vehicle (Veh) control. (*B*) Expression of *Nkd2, Wisp1*, and *Twist1* by qPCR of RNA from lumbar vertebrae (L_4_) relative to *GAPDH*. *n* = 8 to 10/group; *p < .05 versus vehicle (Veh) control; ^†^*p* < .05 versus PTH. (*C*) Luciferase activity (relative light units, RLUs) in UAMS-32P and UMR 106 cells transfected with a TCF-Luc reporter construct. Activity was measured 24 hours after addition of vehicle (0.01% acetic acid), 50 nM PTH(1-34), or 100 ng/mL of sRANKL. *n* = 3/group. *p < .05 versus vehicle.

In view of evidence that sclerostin suppression and stimulation of Wnt signaling promote osteoblast differentiation,(22,23) we wondered whether the smaller pro-osteoblastogenic effect of sRANKL, compared with PTH, was due to the lack of effect of sRANKL on the level of sclerostin. Western blotting of tibial extracts revealed that PTH caused the expected decline in sclerostin, but sRANKL did not (Fig. 5*A*). PTH also increased expression of the canonical Wnt target genes(37) *naked cuticle 2 homologue* (*Nkd2*), *Wnt1 inducible signaling pathway protein 1* (*Wisp1*), and *Twist1* (Fig. 5*B*), but sRANKL had no effect on these transcripts. Interestingly, the degree of PTH-induced suppression of sclerostin in OPG-Fc-pretreated mice was the same as that in controls. However, this response was not associated with increased Wnt signaling, as measured by the expression of the Wnt target genes.

In vitro studies with osteoblastic UAMS-32P and UMR 106 cells showed that PTH increased β-catenin/Tcf-mediated transcription by twofold, as reflected by the activity of a Tcf-*Luciferase* reporter construct (Fig. 5*C*). sRANKL, on the other hand, had no effect.

### PTH, but not sRANKL, increases marrow vascularity

**Fig. 6:**
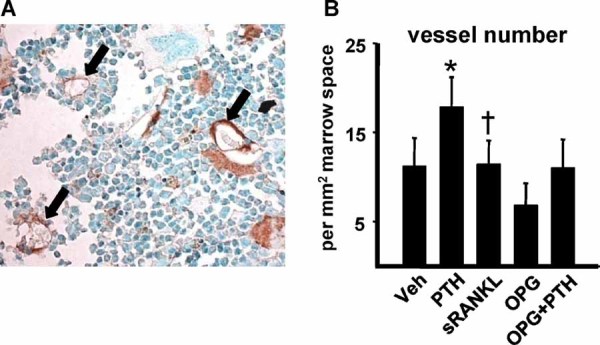
PTH, but not sRANKL, increases marrow vascularity. (*A*) Photomicrograph of blood vessels in the marrow of secondary spongiosa of lumbar vertebrae. Vessels were recognized by CD34^+^ endothelial cells (*arrows*) of vessels containing red blood cells. Original magnification ×200. (*B*) Histomorphometric determination of blood vessels in the marrow space adjacent to the secondary spongiosa. *n* = 4 to 6/group; *p < .05 versus vehicle (Veh) control; ^†^*p* < .05 versus PTH.

Blood vessels were quantified in nondecalcified vertebral bone sections by immunostaining with an anti-CD34 antibody to identify endothelial cells of vessels containing red blood cells (Fig. 6*A*). This assay excludes sinusoidal structures of the bone marrow, which have an irregular shape and lack a continuous layer of CD34^+^ endothelial cells. We found that PTH increased the number of vessels in the bone marrow of the secondary spongiosa by approximately 40% (Fig. 6*B*). Interestingly, neither sRANKL nor OGP-Fc affected vessel number. Moreover, PTH failed to increase blood vessels significantly in mice pretreated with OPG-Fc.

## Discussion

**Fig. 7:**
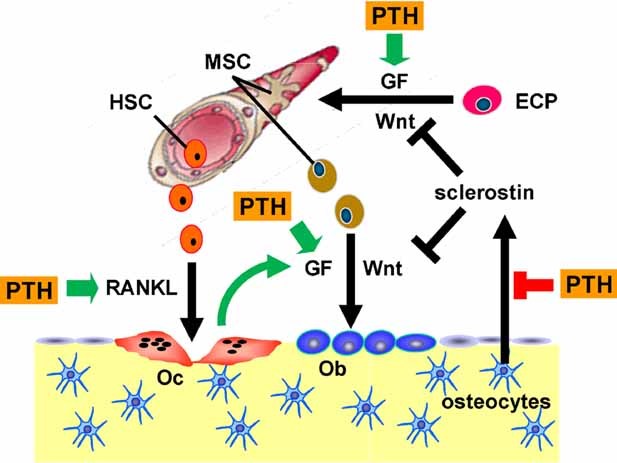
PTH-stimulated osteoclast-dependent and -independent mechanisms involved in the control of osteoblast number during bone remodeling. See text for details. HSC = hematopoietic stem cell progenitors of osteoclasts; Oc = osteoclasts; MSC = mesenchymal stem cell progenitors of osteoblasts; Ob = osteoblasts; ECP = endothelial cell progenitors; GF = growth factors.

The coupling of bone formation to bone resorption during remodeling requires activation of specific pathways that promote the development and survival of osteoblasts. Based on the data presented in this article and previous findings by our laboratory and others, we propose a model to explain how sustained elevation of PTH increases cancellous bone remodeling while maintaining a focal balance between resorption and formation within each BMU (Fig. 7). A sustained rise in PTH increases the local concentration of RANKL and thereby the development of new osteoclasts. Growth factors, such as TGF-β,(11) liberated by osteoclasts from the bone matrix, or cardiotrophin 1 and BMP-6, produced by osteoclasts,(12,13) promote the differentiation and recruitment of osteoblasts to the resorption cavity. However, the number and quality of these osteoblasts are insufficient for complete refilling of the cavity. For this to occur, PTH must activate additional, osteoclast-independent, pathways. Among these, the canonical Wnt signaling pathway appears to be critical.(19) Indeed, PTH activates Wnt signaling by at least three complementary mechanisms: activation of low density lipoprotein receptor-related protein 6 (LRP6),(20) increased synthesis of Wnt ligands,(16) and suppression of sclerostin production by osteocytes.(18,21,22)

Despite the suppression of sclerostin and stimulation of Wnt signaling by continuous elevation of PTH in the experiment described herein, bone mass did not change. This is most likely due to the fact that PTH also stimulated osteoclast differentiation. Hence PTH increased cancellous bone remodeling that remained balanced during this 5-day experiment. The evidence that OPG-Fc pretreatment completely blocks the pro-osteoblastogenic effect of sustained elevation of PTH demonstrates that the RANKL-stimulated osteoclast-dependent mechanism is required for the osteoclast-independent mechanism to become operational. One explanation for this dependency is that the osteoblast progenitors that develop in response to factors released from the bone matrix or from osteoclasts serve as targets for the stimulatory effects of PTH on Wnt signaling via activation of LRP6.(20) These progenitors, as well as mature osteoblasts, may also produce additional pro-osteoblastogenic growth factors in response to PTH. Thus both the osteoclast-dependent and -independent mechanisms may operate in concert to generate and activate enough osteoblasts for complete refilling of the resorption cavity.

The finding that PTH-induced suppression of sclerostin in OPG-Fc-pretreated mice did not lead to increased Wnt signaling and bone formation seems to conflict with evidence that administration of an antisclerostin antibody to ovariectomized rats dramatically increases bone formation.(23) It is also incongruent with evidence that depletion of osteoclasts by pretreatment with the antiresorptive agent alendronate does not prevent the anabolic effect of sclerostin.(38) Although additional studies are needed to resolve this discrepancy, it is possible that complete neutralization of sclerostin by the antibody treatment constitutes a much stronger stimulus to canonical Wnt signaling than the partial depletion of sclerostin afforded by continuous elevation of PTH.

Stimulation of angiogenesis is a second and critical component of the pro-osteoblastogenic effect of continuous PTH elevation (Fig. 7). Like the full osteoblastogenic response, PTH-stimulated angiogenesis requires both osteoclast-dependent and -independent mechanisms. Moreover, the inability of sRANKL to generate enough osteoblasts to refill the resorption cavity is associated with the failure of the cytokine to stimulate angiogenesis. This result adds support to the notion that angiogenesis plays a critical role in osteoblastogenesis. Capillaries may be essential for balanced bone remodeling because of their ability to deliver mesenchymal stem cell progenitors of osteoblasts to the site of bone formation.(26) These progenitors, also known as *pericytes*, reside on the outside surfaces of blood vessels. Pericytes also produce an extracellular matrix and growth factors such as TGF-β that support the development and function of endothelial cells. The dual function of pericytes thus may contribute to the close linkage of angiogenesis and bone formation.

Increased capillaries were noted 25 years ago in bone biopsies from patients with hyperparathyroidism.(39) Moreover, such patients exhibit an increase in the number of circulating endothelial cell progenitors, as defined by their expression of CD45, CD34, and CD31 surface antigens.(40) Interestingly, injections of PTH-related protein (PTHrP) four times a day for 5 days increased angiogenesis in the diploe of 7-week-old mice.(41) Rats given daily injections of PTH also exhibited increased blood vessels near sites of bone formation but no increase in vessel density in the bone as a whole, as revealed by high-resolution µCT imaging.(42) Moreover, intermittent administration of PTH to mice with ischemic heart disease stimulated the migration of endothelial progenitors to and increased neovascularization of the damaged heart tissue.(43) Be that as it may, further studies will be needed to elucidate differences and similarities between the effects of continuous versus intermittent elevation of PTH on blood vessel development in bone and the relationship of new vessels to sites of bone formation.

At this stage, the mechanism by which PTH increases the number of marrow blood vessels is unclear. Besides stimulation of VEGF and angiopoetin 1 synthesis,(27,28) PTH-activated canonical Wnt signaling may be involved because Wnts, TGF-β, and VEGF may act in concert to promote the development of blood vessels.(44,45) PTH-stimulated osteoblastogenesis also could contribute to new blood vessel formation in view of evidence that low oxygen tension, which occurs in osteocytes owing to their distance from blood vessels, stimulates the expression of VEGF by osteoblasts/osteocytes and thereby promotes angiogenesis.(46–48)

PTH may also increase angiogenesis indirectly via stimulation of osteoclastogenesis because osteoclasts have been shown to promote blood vessel formation by producing matrix metalloproteinase 9, which releases growth factors from the extracellular matrix.(41) As we observed, OPG prevented the proangiogenic effect of PTHrP in explanted fetal metatarsal bone.(41) However, in contrast to our findings, sRANKL stimulated angiogenesis when injected subcutaneously over calvaria of growing 7-week-old mice four times per day.(41) This seeming discrepancy most likely reflects methodologic differences between the two studies, including local versus systemic administration of sRANKL, calvaria versus the axial skeleton, and/or the age of the animals.

Despite the failure of sRANKL to generate an adequate number of active osteoblasts to refill the resorption cavity, those that did develop exhibited a low prevalence of apoptosis. Hence the initial osteoblastogenic response mediated by osteoclasts was magnified by prolongation of osteoblast lifespan. In situ hybridization studies have shown that RANK is not expressed by osteoblasts in vivo.(49) Thus the antiapoptotic actions of sRANKL on osteoblasts probably are mediated indirectly by release of factors from the bone matrix or from osteoclasts. In support of this notion, TGF-β, an established inhibitor of osteoblast apoptosis,(50) is released from the bone matrix in vivo and in vitro.(11) As shown in this and an earlier study of ours,(30) continuously elevated PTH has no effect on osteoblast apoptosis in vivo. Thus indirect pro-survival effects of RANKL on osteoblasts in the hyperparathyroid state may be counterbalanced by direct or indirect proapoptotic actions of the hormone. In support of this notion, we have demonstrated previously that more than 6 hours of exposure of osteoblastic cells to PTH suppresses the synthesis of Runx2,(30) which is required for the pro-survival effects of the hormone. In addition, Chen and colleagues have reported that long-term culture of osteoblastic cells with PTH stimulates osteoblast apoptosis.(51)

The antiapoptotic effect of sRANKL on osteoblasts raises the possibility that OPG-Fc stimulates apoptosis. We were unable to measure the prevalence of apoptotic osteoblasts in mice receiving OPG-Fc because of the paucity of osteoblasts. However, the relatively slow decline in osteoblasts following administration of OPG-Fc, together with the demonstration that OPG-Fc increased BMD, indicates that viable osteoblasts refilled the resorption cavities in OPG-Fc-treated mice. In contrast, OPG-Fc caused a rapid decline in osteoclast number, as would be expected by the loss of the pro-differentiating and pro-survival effects of RANKL on osteoclasts. Administration of alendronate to mice has the same effect as OPG-Fc on vertebral osteoclast and osteoblast number, as well as cancellous bone volume.(52) Thus, at this stage, the evidence does not support the hypothesis that OPG-Fc stimulates osteoblast apoptosis.

In summary, we have shown that PTH-stimulated bone remodeling maintains a focal balance between bone resorption and bone formation in cancellous bone by increasing both osteoclasts and osteoblasts. We propose that the latter occurs in part via a RANKL-stimulated osteoclast-mediated component. However, complete refilling of the resorption cavity requires additional actions of PTH that unleash canonical Wnt signaling and stimulate angiogenesis. Future advances in our understanding of the mechanisms responsible for balanced versus unbalanced remodeling will require elucidation of the mechanism(s) by which Wnt signaling and other factors induced by PTH promote the proliferation, differentiation, and recruitment of osteoblasts to the BMU, as well as investigation of the role of angiogenesis in the regulation of bone remodeling.
